# Optimizing acute stroke treatment process: insights from sub-tasks durations in a prospective observational time and motion study

**DOI:** 10.3389/fneur.2023.1253065

**Published:** 2023-10-27

**Authors:** Gizem Koca, Mukesh Kumar, Gord Gubitz, Noreen Kamal

**Affiliations:** ^1^Department of Industrial Engineering, Faculty of Engineering, Dalhousie University, Halifax, NS, Canada; ^2^Division of Neurology, QEII – Halifax Infirmary (HI) Site, Nova Scotia Health, Halifax, NS, Canada; ^3^Division of Neurology, Department of Medicine, Dalhousie University, Halifax, NS, Canada; ^4^Department of Community Health and Epidemiology, Faculty of Medicine, Dalhousie University, Halifax, NS, Canada

**Keywords:** acute ischemic stroke, thrombolysis, endovascular thrombectomy (EVT), stroke pathways, delay factors, observational time study, emergency department (ED)

## Abstract

**Background:**

Rapid treatment is critical in managing acute ischemic stroke (AIS) to improve patient outcomes. Various strategies have been used to optimize this treatment process, including the Acute Stroke Protocol (ASP) activation, and minimizing the duration of key performance metrices, such as door-to-needle time (DNT), CT-to-needle time (CTNT), CT-to-groin puncture time (CTGP), and door-to-groin puncture time (DGPT). However, identifying the delay-causing sub-tasks within the ASP could yield novel insights, facilitating optimization strategies for the AIS treatment process.

**Methods:**

This two-phase prospective observational time and motion study aimed to identify sub-tasks and compare their respective durations involved in the treatment process for AIS patients within ASPs. The study compared sub-task durations between “routine working hours” and “evenings and weekends” (after-hours), as well as between stroke neurologists and non-stroke neurologists. Additionally, the established performance metrices of AIS were compared among the aforementioned groups.

**Results:**

Phase 1 identified and categorized 34 sub-tasks into five broad categories, while Phase 2 analyzed the ASP for 389 patients. Among the 185 patients included in the study, 57 received revascularization treatment, with 30 receiving intravenous (IV) thrombolysis only, 20 receiving endovascular thrombectomy (EVT) only, and 7 receiving both IV thrombolysis and EVT. Significant delays were observed in sub-tasks including triage, registration, patient history sharing, treatment decisions, preparation of patients, preparation of thrombolytic agents, and angiosuite preparation. The majority of these significant delays (*P* < 0.05) were observed when were performed by a non-stroke neurologist and during after-hours operations. Furthermore, certain sub-tasks were exclusively performed during after-hours or when the treatment was provided by a non-stroke neurologist. Consequently, DNT, CTNT, and CTGP were significantly prolonged for both non-stroke neurologists and off-hours treatment. DGPT was significantly longer only when the ASP was conducted by non-stroke neurologists.

**Conclusions:**

The study identified several sub-tasks that lead to significant delays during the execution of the ASP. These findings provide a premise to design targeted quality improvement interventions to optimize the ASP for these specific delay-causing sub-tasks, particularly for non-stroke neurologists and after-hours. This approach has the potential to significantly enhance the efficiency of the AIS treatment process.

## 1. Introduction

Providing rapid and efficient treatment is crucial for managing acute ischemic stroke (AIS) and improving patient outcomes. Quality improvement studies in AIS have focused on identifying, establishing, and improving key process indices, including door-to-CT (DTCT), door-to-needle time (DNT), CT-to-needle time (CTNT), CT-to-groin puncture time (CTGP), and door-to-groin puncture time (DGPT) (1–9). These indices serve as benchmarks for evaluating and optimizing the efficiency of an Acute Stoke Protocol (ASP) to ensure optimal numbers of patients have good outcomes. The Canadian Best Practice Recommendations for Acute Stroke recommend DNT and DGPT benchmarks of 30 and 60 minutes, respectively (10, 11). Significant efforts to improve the treatment process of AIS has been made in Canada and other parts of the world (1–9, 12–15). However, the AIS treatment process is inherently intricate and multifaceted, involving numerous interdependent processes and clinical specialties. This intricate composition makes the process susceptible to potential delays, necessitating a continuous pursuit of optimization to ensure the achievement of optimal patient outcomes.

A novel approach could be to break-down the AIS treatment process into smaller steps, which we will refer to as “sub-tasks”. In healthcare, no formal definition for “sub-tasks” exists, but they can be seen as small, specific actions or components within a larger task or process. These sub-tasks contribute to the overall completion of the task and can be identified, measured, and optimized to make the process more efficient. Within the context of the AIS treatment process, key tasks that is used in studies aimed to lower treatment times include DTCT, CTNT, and CTGP times. These tasks can then be broken down into smaller tasks (sub-tasks) such as patient triage, registration, blood sample collection, sharing medical history, neurological evaluation, and transport to the radiology department, among others. The approach of identifying sub-tasks and quantifying their durations, followed by the identification of sub-tasks resulting in delays may provide valuable insights into the AIS treatment process, which may inform new improvement strategies and ultimately lead to better patient outcomes.

Observational “time and motion study” is a method from industrial engineering that may aid in identifying and documenting sub-tasks and their durations. This method involves observing and measuring tasks within a process using a stopwatch to establish the corresponding times for the sub-tasks. Observational time and motion studies have previously been utilized to enhance overall emergency department (ED) workflow and minimize wait times (16–18). However, its application in optimizing the AIS treatment process remains unexplored.

The objectives of this study encompassed three main aspects. Firstly, it aimed to identify sub-tasks involved in the AIS treatment process. Secondly, it measured and recorded the durations of these sub-tasks to further determine potential sub-tasks causing treatment delays. This objective involved comparing sub-task durations between “routine working hours” and “evenings and weekends” (after-hours), as well as between stroke neurologists and non-stroke neurologists. Finally, the study aimed to assess the impact of these delay-causing sub-tasks on key process metrices of the ASP process, such as DNT and DGPT, among the abovementioned groups.

## 2. Materials and methods

A two-phase prospective observational time and motion study was undertaken to evaluate the AIS treatment process. The research protocol of this study received an exemption from the Nova Scotia Health Authority Research Ethical Board due to its focus on quality improvement. Instead, the study was approved by the Nova Scotia Health Authority Quality Improvement and Safety Council. Since the study was a quality improvement initiative and did not collect patient-level data, consent was not required from patients or their next of kin, either in written or verbal form.

### 2.1. Study center and population

The study was conducted in the Emergency Department (ED) at the Queen Elizabeth II Health Science Center (QEII) in Halifax, Nova Scotia (NS), Canada. QEII is the only comprehensive stroke center in the province of Nova Scotia. This hospital offers intravenous (IV) thrombolysis treatment to patients within its catchment area and provides endovascular thrombectomy (EVT) treatment to patients residing in the provinces of NS and Prince Edward Island (PE). Both NS and PE are relatively small in terms of area and population, with NS having a population of one million and PEI having a population of 173,000. QEII is a large urban tertiary care teaching hospital. The center was staffed with two stroke neurologists and 19 non-stroke neurologists during the study period, and has 24/7 on-site CT (computed tomography) services, along with interventional neuroradiologists.

The study focused on adult patients who were presented at the ED with an acute suspected stroke, resulting in the activation of the ASP. The study included only patients who had ASP activation before their arrival. Patients who had an in-hospital stroke and those who arrived within 10 min of pre-notification were not eligible. These exclusions were due to the unavailability of the on-site observer, who had to reach the location after receiving the pre-notification. The patient population encompassed those with ischemic stroke, hemorrhagic stroke, transient ischemic attack (TIA), or stroke mimic.

### 2.2. Standard acute stroke treatment process at the study center

The AIS treatment process begins with the ASP activation by either the attending neurologist or the ED charge physician, either before or after the patient's ED arrival. A multidisciplinary stroke team, including a neurologist, neurology resident, stroke nurse (during routine hours), CT technician, and radiologist/radiology resident, is notified during this pre-notification period using a pager notification system. In the ED triage area, the patient is assessed by the ED nurse, neurologist, and neurology resident. Neurological examination, National Institutes of Health Stroke Score (NIHSS) assessment, and data collection from paramedics ensue.

The patient is then transported to the CT Scanner for brain imaging, starting with a plain CT head potentially followed by CT angiography (CTA), and CT perfusion (CTP). After imaging, patients may be further evaluated in the Diagnostic Radiology Department or the ED, depending on the neurologist's decision. Upon AIS confirmation, eligibility for IV thrombolysis and EVT is determined. While consent may be sought, emergency consent is typically applicable per Canadian guidelines. Treatment pathways vary based on IV thrombolysis, EVT, or both, and the time of day. During routine hours, IV thrombolysis occurs in the Radiology Department, while EVT patients await their turn in the Interventional Radiology Department angiosuite. After hours, patients return to the ED for IV thrombolysis and then proceed to the angiosuite for EVT, unless urgent issues necessitate ED treatment.

### 2.3. Observation and data collection phases

All patients were observed between 7 am and 11 pm, with “routine working hours” observations conducted on weekdays from 8 am to 4 pm, and all other observations considered during “after-hours”. The rationale for considering the observation time from 7 am to 11 pm stemmed from the limited availability of the only observer, who meticulously documented the execution of each ASP.

#### 2.3.1. Phase 1 observations to identify sub-tasks

In phase 1 of this study, conducted from November 1, 2021 to November 16, 2021, a single observer closely observed the AIS treatment process carried out by the stroke team. The observer, GK, is a Ph.D. candidate affiliated with a healthcare optimization laboratory dedicated to enhancing AIS treatment delivery. The observer aimed primarily to identify and document the sub-tasks within the AIS treatment process. Sub-tasks were identified based on reviews and opinions of stroke experts. Initially, five major tasks of the treatment process were defined based on the current literature: (1) Arrival to Imaging (Prior to Imaging), (2) Image Acquisition, (3) Treatment Decision, (4) Preparations for Thrombolysis, and (5) Preparation for EVT.

“Arrival to imaging” included the temporal duration from the suspected AIS patient's arrival at the ED to their transfer to the radiology department for imaging.

“Image acquisition” denoted the interval commencing upon arrival at the radiology department and culminating in the acquisition of CT imaging.

“Treatment decision” included the temporal duration between the acquisition of CT imaging and the determination to initiate thrombolytic therapy, based on CT imaging and AIS treatment guidelines.

“Preparations for thrombolysis” referred to the duration extending from the treatment decision to the actual administration of thrombolytic therapy.

“Preparation for EVT” signified the time elapsed from the administration of thrombolytic therapy to the initiation of groin puncture for the purpose of performing EVT.

Finally, in this phase of our study, sub-tasks carried out to complete each of these major tasks were meticulously identified and documented, relying upon the reviews and insights of stroke experts.

#### 2.3.2. Phase 2 observations to measure and document the durations of sub-tasks

During the second observation phase—November 17, 2021 to June 15, 2022, the ASP workflow was closely monitored by the same observer to document all sub-tasks and their relevant information on a standard treatment process report form. The observer shadowed the workflow and recorded the following variables:

- *Mode of arrival*: whether the patient arrived using EMS or as a walk-in.
- *Type of neurologist*: if the ASP was conducted by a stroke neurologist or a non-stroke neurologist.
- *Time of day*: whether the patient arrived during routine working hours or after-hours.
- *Diagnosis*: determined after CT image acquisition, categorized as AIS, hemorrhage, stroke mimic, or unknown.
- *Treatment types*: whether the patient received IV thrombolysis, EVT or both.
- *Start and end times of each sub-task*: recorded for each suspected AIS patient.

If any new sub-tasks were observed during this phase, they were documented and recorded, along with any interruptions.

### 2.4. Outcomes

The outcome of the phase 1 study involved the identification and documentation of all the sub-tasks necessary to complete each major task within the ASP. The phase 2 study outcomes included differences in median durations of sub-tasks, comparing routine working hours with after-hours and stroke neurologists with non-stroke neurologists. Additionally, the study outcomes for the key process metrices of the ASP included differences in median durations of process metrices, including DTCT, CTNT, CTGP, DNT, and DTGP times.

### 2.5. Statistical analysis

Descriptive statistics were used to describe the baseline characteristics of the participants. Also, the baseline characteristics were compared between routine working hours and after-hours as well as between non-stroke neurologists and stroke neurologists, and the Chi-Square test was employed to analyze these categorical variables. Further, for each sub-task, the median duration and Interquartile Range (IQR) were calculated, and comparisons were made between the aforementioned groups using the Mann-Whitney U Test. Finally, the study also calculated the median DTCT, CTNT, CTGP, DNT, and DTGP time, with comparisons made between the above-mentioned groups using the Mann-Whitney U Test. These final comparisons were carried out to gain a comprehensive understanding of the effects of sub-tasks on the key process metrices of the ASP. The statistical analyses were conducted using Minitab version 21 (State College, PA).

## 3. Results

### 3.1. Phase 1

#### 3.1.1. Observations to identify sub-tasks

During the phase 1 observational study, we identified the sub-tasks involved in the ASP guiding the treatment of AIS. Our observations led us to categorize the steps during ASP flow into five primary steps: *Arrival to Imaging* (Prior to Imaging), *Image Acquisition, Treatment Decision, Preparations for Thrombolysis*, and *Preparation for EVT*. We further classified the sub-tasks within each category based on their order of occurrence and location. In addition to the sub-tasks identified during Phase 1, some were also observed during Phase 2, resulting in a total of 34 sub-tasks related to ASP being recorded at the study center. Some sub-tasks were merged due to patient-related reasons, hospital staff-related factors, or study center-related constraints. This combining of sub-tasks was considered beneficial for assessment and comparison purposes.

**Figure 1:**
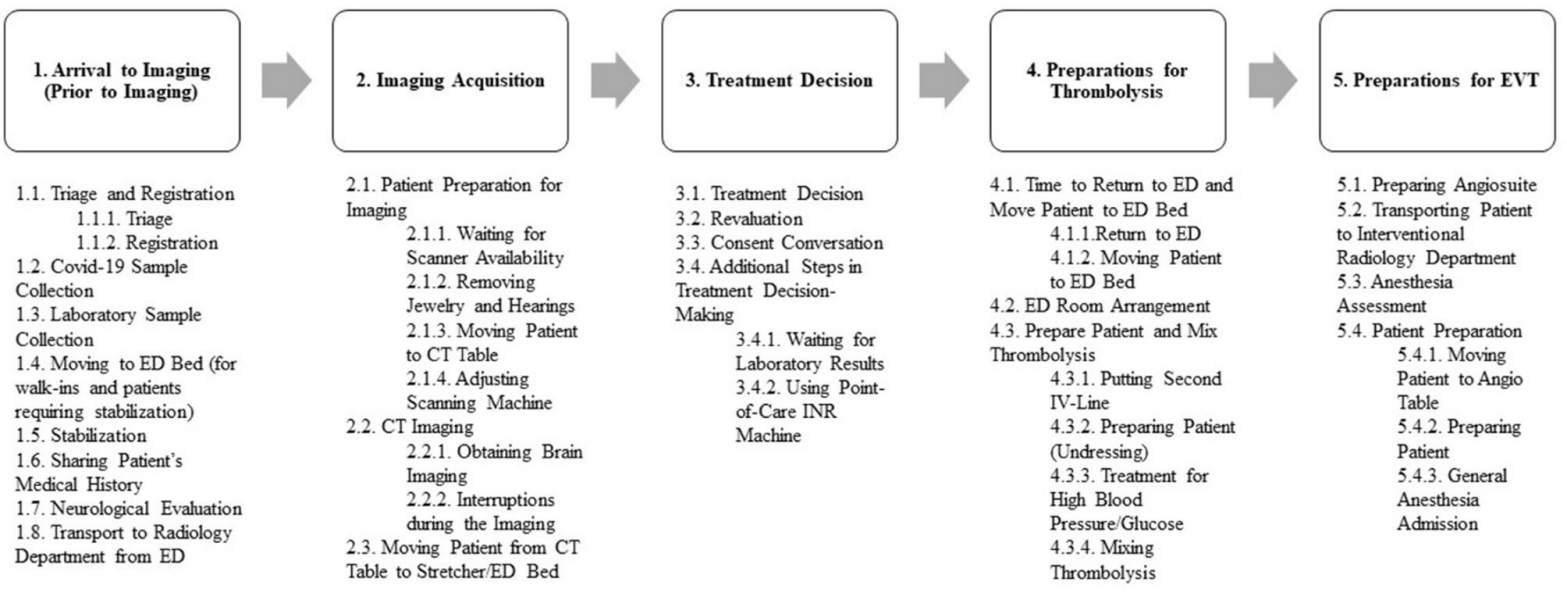
The sub-tasks identified during the observations.

Figure 1 depicts all the sub-tasks that took place during ASP process, and Supplementary Table 1 provides a detailed explanation and definition of each sub-task.

### 3.2. Phase 2

#### 3.2.1. Baseline characteristics of the observed patients and their treatment process

**Figure 2:**
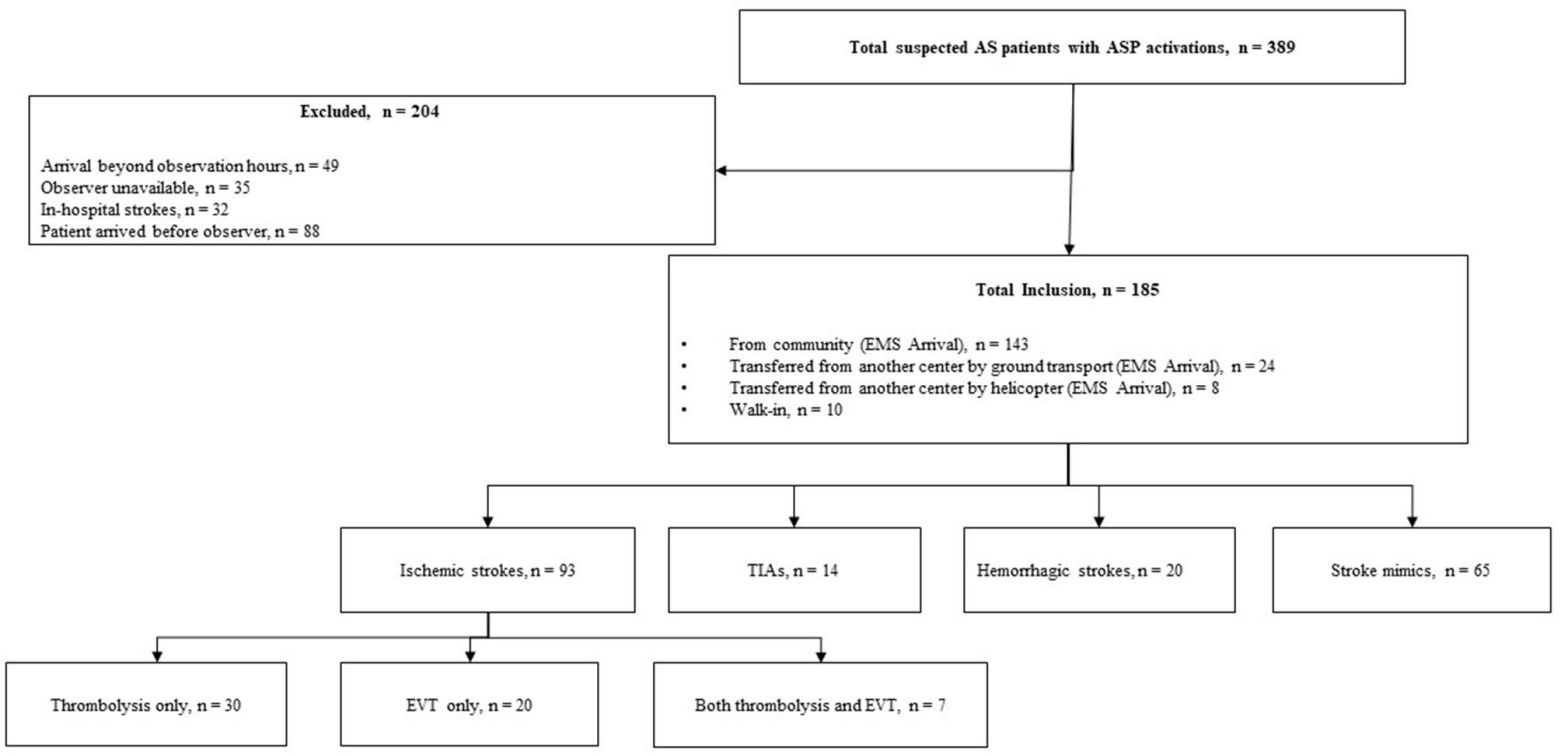
Flowchart of suspected acute stoke patients arrived with ASP activation.

A total of 389 patients with suspected AIS and an ASP activation were assessed. Amongst them, 204 (52.4%) were excluded due to various reasons as mentioned in Figure 2. As a result, 185 (47.6%) patients with suspected AIS were observed during the study. Among the included patients, 106 (57.3%) arrived after-hours, 175 (94.6%) arrived by ground ambulance, 139 (75.1%) were assessed by a non-stroke neurologist, and 120 (64.9%) were diagnosed with stroke.

Out of the 120 patients diagnosed with stroke, 93 (77.5%) had AIS, 14 (11.7%) had TIAs, and 13 (10.8%) had hemorrhagic strokes. Out of the 65 patients who did not have a stroke diagnosis, 3 (4.6%) were diagnosed with migraine, 5 (7.7%) were diagnosed with seizure, and 57 (87.7%) had an unclear diagnosis even after CT imaging; they were subsequently transferred to the ED for further assessment. Of the 93 patients diagnosed with ischemic stroke, 57 (61.3%) received treatment at the study center. The treatment modalities were distributed as follows among the treated patients: IV thrombolysis only (30 patients, 52.6%), EVT only (20 patients, 35.1%), and both IV thrombolysis and EVT (7 patients, 12.3%).

Additional information regarding the baseline characteristics of the observed patients and their treatment process, categorized based on both the time of day and the assessing neurologist, can be explored in Table 1.

**Table 1:** Comparison of baseline characteristics based on the time of arrival and assessing neurologist.

**Patient and treatment characteristics**	**Comparison based on the time of arrival**			**Comparison based on the assessing neurologist**		
	Routine working hours^*^(***n*** = **79)**	After-hours^**^(***n** =* **106)**	***P-*** **value**	**Non-stroke neurologist (*****n** =* **139)**	**Stroke neurologist (*****n** =* **46)**	***P-*** **value**
**Analysis of all study participants (*****n** =* **185)**						
**EMS transferred**, ***n*** **(%)**	71 (89.9)	104 (98.1)	**0.014**	131 (94.2)	44 (95.7)	0.714
**Assessed by stroke neurologist**, ***n*** **(%)**	36 (45.6)	10 (9.6)	**< 0.001**			
**Patients diagnosed with stroke**, ***n*** **(%)**	54 (68.4)	66 (62.3)	0.391	89 (64.0)	31 (67.4)	0.679
**Patients diagnosed with ischemic stroke only**, ***n*** **(%)**	39 (49.4)	54 (50.9)	0.832	72 (51.8)	21 (45.7)	0.470
**Received treatment**, ***n*** **(%)**	27 (34.2)	30 (28.3)	0.392	41 (29.5%)	16 (34.8)	0.501
	Routine working hours^*^(***n** =* **39)**	After-hours^**^(***n** =* **54)**	***P-*** **value**	**Non-stroke neurologist (*****n** =* **72)**	**Stroke neurologist (*****n** =* **21)**	***P-*** **value**
**Analysis of patients diagnosed with ischemic stroke only (*****n** =* **93)**						
**Received treatment**, ***n*** **(%)**	27 (69.2)	30 (55.6)	0.182	41 (56.9)	16 (76.2)	0.111
	Routine working hours^*^(***n** =* **27)**	After-hours^**^(***n** =* **30)**	***P-*** **value**	**Non-stroke neurologist (*****n** =* **41)**	**Stroke neurologist (*****n** =* **16)**	***P-*** **value**
**Analysis of patients who received treatment only (*****n** =* **57)**						
**Type of treatment**, ***n*** **(%)**			0.675			1.000
Thrombolysis only, *n* (%)	15 (55.6)	15 (50.0)		22 (53.7)	8 (50.0)	
EVT only, *n* (%)	8 (29.6)	12 (40.0)		14 (34.1)	6 (37.5)	
Both thrombolysis and EVT, *n* (%)	4 (14.8)	3 (10.0)		5 (12.2)	2 (12.5)	

Bold values show the statistical significance.

* Weekdays, 8 am–4 pm.

** Weekdays 7 am–8 am and Weekends, 4 pm–11 pm.

#### 3.2.2. The delay-causing sub-tasks

Table 2 and Supplementary Table 2 presents a comprehensive overview of the sub-tasks, including the median duration of each sub-task, the frequency of sub-task execution among included patients, and a comparison between routine working hours vs. after-hours and stroke neurologist vs. non-stroke neurologist groups. Below a description of the delay-causing sub-tasks is provided, which were identified based on the time of day and type of neurologist. The results are presented in a sequence that is consistent with the progression of the ASP.

**Table 2:** Comprehensive overview of the sub-tasks observed during the study.

**Sub-tasks**	**Median time (IQR)**	**Routine working hours vs. after-hours**			**Stroke neurologist vs. non-stroke neurologist**		
		**Routine working hours**	**After-hours**	***P-*** **value**	**Stroke neurologist**	**Non-stroke neurologist**	***P-*** **value**
**1. Arrival to imaging (prior to imaging)**							
1.1. Time to triage and registration	3 (2–7) *n =* 159	2 (1–7) *n =* 71	4 (2–7) *n =* 88	**0.049**	2 (2–6.75) *n =* 44	3 (2–8) *n =* 115	0.067
1.2. Covid-19 sample collection time	1 (0–1) *n =* 84	1 (0–1) *n =* 39	1 (0–1.5) *n =* 45	0.215	0 (0–4) *n =* 19	1 (0–1) *n =* 65	0.481
1.3. Laboratory sample collection time	3 (2.75–5) *n =* 102	3 (3–5) *n =* 48	3.5 (2–5) *n =* 54	0.833	4 (3–6) *n =* 27	3 (2–5) *n =* 75	0.173
1.4. Time to move to ED bed (for walk-ins and patients requiring stabilization)	1 (1–1.5) *n =* 9	1 (1–1.5) *n =* 5	1 (1–1.75) *n =* 4	1	1 (1–2) *n =* 3	1 (1–1.25) *n =* 6	0.722
1.5. Time for stabilization	33 (3–45) *n =* 3	24 (–) *n =* 2	33 (–) *n =* 1	-	45 (–) *n =* 1	18 (–) *n =* 2	-
1.6. Time to share patient's medical history	2 (1–2) *n =* 115	2 (1–2) *n =* 48	2 (1–2) *n =* 67	0.883	1.5 (1–2) *n =* 28	2 (1–2) *n = 87*	**0.039**
1.7. Time for neurological evaluation	3 (2–5) *n =* 156	3 (2–5) *n = 67*	3 (2–5) *n = 89*	0.453	3 (2–4) *n = 41*	3 (2–5) *n = 115*	0.523
1.8. Time to transport to radiology department from ED	2 (2–2) *n =* 168	2 (2–2) *n = 72*	2 (2–2) *n = 96*	0.803	2 (2–2) *n = 44*	2 (2–2) *n = 124*	0.874
**2. Imaging acquisition**							
2.1. Time to prepare patient for imaging	4 (4–5) *n =* 179	4 (3.5–5) *n = 77*	4 (4–6) *n = 102*	0.154	4 (4–5) *n = 45*	4 (4–5) *n = 134*	0.999
2.2. Time to CT imaging	6 (5–7) *n =* 182	6 (5–7) *n = 79*	6 (5–8) *n = 106*	0.232	6 (5–7) *n = 46*	6 (5–8) *n = 139*	0.326
2.3. Time to move patient from CT table to stretcher/ED bed	2 (2–3) *n =* 173	2 (2–3) *n = 73*	2 (2–3) *n = 100*	0.797	2 (1–2.75) *n = 44*	2 (2–3) *n = 129*	0.122
**3. Treatment decision**							
3.1. Treatment decision time	7 (4–11.25) *n =* 54	5 (2–10) *n =* 27	9 (6–12) *n =* 27	**0.019**	4.5 (0.5–9.75) *n =* 16	8 (5–12.25) *n =* 38	**0.029**
3.2. Time to revaluation	3 (2.5–8) *n =* 9	5.5 (–) *n =* 2	3 (2–8) *n =* 7	0.661	1 (–) *n =* 1	3 (3–8) *n =* 8	-
3.3. Time to consent conversation	2 (2–3) *n =* 24	2 (2–2.75) *n =* 8	2 (1.25–3) *n =* 16	0.878	2 (2–2.5) *n =* 5	2 (1–3) *n =* 19	0.887
3.4. Time for additional steps in treatment decision-making	12.5 (2.8–93.3) *n* = 6	6 (2–94) *n =* 3	19 (3–93) *n =* 3	1	(–) *n = 0*	12.5 (2.8–93.3) *n =* 6	-
**4. Preparations for thrombolysis**							
4.1. Time to return ED and to move patient to ED bed	3 (3–4) *n =* 25	3 (2–4) *n =* 7	3 (3–4) *n =* 18	0.823	3.5 (2.25–7) *n =* 4	3 (3–4) *n =* 21	0.784
4.2. Time for ED room arrangement	4 (1–6.5) *n =* 9	7 (–) *n =* 1	3 (1–5.5) *n =* 8	-	(–) *n = 0*	4 (1–6.5) *n =* 9	-
4.3. Time to prepare patient and to mix thrombolysis	7 (3–12) *n =* 37	4 (3–7) *n =* 19	11 (6.5–16) *n =* 18	**0.004**	3 (3–6) *n =* 10	8 (5–13) *n =* 27	**0.011**
**5. Preparations for EVT**							
5.1. Time to prepare angiosuite	34 (26–47.5) *n =* 25	27.5 (23.75–32.5) *n = 10*	45 (33–50) *n = 15*	**< 0.001**	27.5 (23.25–31.75) *n = 8*	43 (31–50) *n = 17*	**0.012**
5.2. Time to transport patient to interventional radiology department	2 (1–3) *n =* 23	2 (1.25–2) *n = 12*	2 (1–3) *n = 11*	0.248	2 (1.25–2) *n = 8*	2 (1–3) *n = 15*	0.253
5.3. Anesthesia assessment time	2 (1–4) *n =* 15	2 (1–2.5) *n = 5*	3 (1.75–5.25) *n = 10*	0.150	2 (1–2) *n = 3*	3 (1.25–4.75) *n = 12*	0.248
5.4. Patient preparation time	12 (9.5–21.5) *n =* 25	14.5 (10.25–30) *n = 12*	10 (9–19.5) *n = 13*	0.201	14.5 (10.75–30) *n = 8*	11 (9–19.5) *n = 17*	0.220

Bold values show the statistical significance.

During the *arrival to imaging stage*, it was observed that sub-tasks “triage and registration” (median time in minutes, 2 vs. 4; *U* = 2,560.5; *P* = 0.049; *Z* = −1.97) took significantly longer time during off-hours, while sub-tasks “sharing patient's medical history” (median time in minutes, 1.5 vs. 2; *U* = 923.0; *P* = 0.038; *Z* = −2.07) took longer when the attending was a non-stroke neurologist. Conversely, during the *image acquisition stage*, none of the sub-tasks caused delays in treatment process, regardless of the time of day or the attending neurologist.

During the *treatment decision stage*, the sub-task “treatment decision time” took significantly longer both during off-hours (median time in minutes, 5 vs. 9; *U* = 228.0; *P* = 0.019; *Z* = −2.37) and when the ASP was carried out by a non-stroke neurologist (median time in minutes, 4.5 vs. 8; *U* = 188.5; *P* = 0.029; *Z* = −2.19). Further, the frequencies of sub-tasks “re-evaluation of the treatment decision,” “consent conversation,” and “laboratory results wait” were skewed toward non-stroke neurologists (88.9, 79.2, and 100.0%, respectively), and it was observed that stroke neurologists typically do not perform these sub-tasks. However, these sub-tasks did not reach a statistical significance due to them being performed in small numbers by stroke neurologists.

During the *preparations for thrombolysis stage*, the sub-task “preparing patient and mixing thrombolytic agent” took significantly longer both during off-hours (median time in minutes, 4 vs. 11; *U* = 76.0; *P* = 0.004; *Z* = −2.91) and when the attending was a non-stroke neurologist (median time in minutes, 3 vs. 8; *U* = 60.5; *P* = 0.010; *Z* = −2.57). Further, the sub-task “ED room arrangement” was exclusively observed during non-stroke neurologist treatment, and the majority (88.9%) of these arrangements occurred after-hours. Again, statistical significance cannot be established for this sub-task due to skewed distribution of the frequencies among the groups.

During the *preparations for EVT stage*, the sub-task “preparing angiosuite” took significantly longer both during off-hours (median time in minutes, 27.5 vs. 45; *U* = 24.5; *P* = 0.005; *Z* = −2.80) and when the attending was a non-stroke neurologist (median time in minutes, 27.5 vs. 43; *U* = 24.5; *P* = 0.011; *Z* = −2.54).

#### 3.2.3. Comparison of the key process metrices of ASP process

In this study, the median times for overall key process metrices of the ASP process were determined. The median DTCT time was 14 min (IQR, 12–18.25), CTNT was 24 min (IQR, 14.5–35.5), DNT was 37 min (IQR, 29–50.5), CTGP time was 61.5 min (IQR, 52.5–91.25), and DTGP time was 74 min (IQR, 58–95). Further, group-wise comparisons of these process metrices were performed. For the routine working hours vs. after-hours comparisons, CTNT (median time in minutes, 15 vs. 27; *U* = 61.5; P < 0.001; *Z* = −3.33), DNT (median time in minutes, 30 vs. 42; *U* = 83.5; *P* = 0.008; *Z* = −2.66), and CTGP time (median time in minutes, 51.5 vs. 72; *U* = 35.5; *P* = 0.035; *Z* = −2.11) were significantly longer during after-hours. Similarly, CTNT (median time in minutes, 12.5 vs. 25; *U* = 55.5; *P* = 0.006; *Z* = −2.72), DNT (median time in minutes, 27 vs. 40; *U* = 56.5; *P* = 0.007; *Z* = −2.69), CTGP time (median time in minutes, 49.5 vs. 70; *U* = 21.5; *P* = 0.009; *Z* = −2.60), and DTGP time (median time in minutes, 59 vs. 83; *U* = 37.0; *P* = 0.038; *Z* = −2.07) were significantly longer when a non-stroke neurologist carried out the ASP. Table 3 provides a detailed summary of the outcomes of all key process metrices in the ASP process for this study.

**Table 3:** Summary of all the established key process metrices in the ASP process for the current study.

	**Overall median time (IQR)**	**Routine working hours vs. after-hours (min)**			**Stroke neurologist vs. non-stroke neurologist (min)**		
		**Routine working hours**	**After-hours**	***P-*** **value**	**Stroke neurologist**	**Non-stroke neurologist**	***P-*** **value**
Door-to-CT time (DTCT)	14 (12–18.25) *n =* 182	13 (11–19) *n =* 79	14 (12–18) *n =* 103	0.899	13.5 (10–18) *n =* 46	14 (12–19) *n =* 136	0.155
CT-to-needle time (CTNT)	24 (14.5–35.50) *n =* 37	15 (11–24) *n =* 19	27 (24–42) *n =* 18	**< 0.001**	12.5 (9–22) *n =* 10	25 (20–38) *n =* 27	**0.006**
Door-to-needle time (DNT)	37 (29–50.50) *n =* 37	30 (23–50) *n =* 19	42 (37–56) *n =* 18	**0.008**	27 (20.5–39.25) *n =* 10	40 (34–65) *n =* 27	**0.007**
CT-to-groin puncture time (CTGP)	61.5 (52.5–91.25) *n =* 24	51.5 (41.25–66) *n =* 12	72 (55.5–91.25) *n =* 12	**0.035**	49.5 (40–60.75) *n =* 8	70 (52.5–91.25) *n =* 16	**0.009**
Door-to-groin puncture time (DTGP)	74 (58–95) *n =* 27	68 (53.8–80.8) *n =* 12	85 (58–102) *n =* 15	0.213	59 (49–74) *n =* 8	83 (59–102) *n =* 19	**0.038**

Bold values show the statistical significance.

## 4. Discussion

This study shows that applying an observational time and motion study to first identify and then quantify tasks has value in recognizing areas for improvement to reduce treatment time for AIS patients. Time and motion studies have been used in emergency departments to better understand workflow (17, 19–21); however, time and motion study has not been applied to AIS treatment. The US Get With The Guidelines-Stroke registry collected whether specific predetermined delays occurred such as determining eligibility and hypertension control; however, the time spent on these tasks were not evaluated (3).

This study identified and quantified each specific task or *sub-task* in a single center, which is likely the most appropriate level to evaluate processes using time and motion studies. Each center often develops its own unique processes that are difficult to generalize across various centers. Therefore, the use of time and motion studies to identify areas for improvement can be extended to other individual centers, including smaller community and rural hospitals, which present distinct challenges, particularly concerning on-site resources and the need for tele-stroke services to make treatment decisions (22).

The results of this time and motion study showed that the study center had significant delays during off-hours. Poor patient outcomes (23, 24) and workflow delays (25) during off-hours have been observed in EVT treatment. This study provides and reaffirms the occurrence of these delays during off-hours at the study center, affecting both thrombolysis and EVT treatment. Furthermore, this study highlights specific tasks contributing to treatment delays during off-hours, such as treatment decision-making, preparing patient and mixing the lytic drug, ED room arrangement before IV thrombolysis (89% occurrences during off-hours), and preparing the angiosuite.

Additionally, this time and motion study revealed that non-stroke neurologists performed additional non-value-added tasks and took longer task completion times in comparison to their stroke-trained colleagues. Specifically, non-stroke neurologists took longer in obtaining patient history and making treatment decisions. Further, specific sub-tasks, such as the re-evaluation of the treatment decision, consent conversation, laboratory results wait, and ED room arrangement, were either exclusively conducted or disproportionately performed by non-stroke neurologists, resulting in extended treatment times. This result is important, as there are currently very few studies that show that stroke trained neurologists perform more efficiently in the treatment of AIS patients. Challenges in the recruitment and retention of stroke neurology workforce has been identified (26), which is likely even more prevalent in the study setting of Nova Scotia, an economically constrained Canadian province with a small population. Furthermore, the study center is in a relatively small city of approximately 350,000 people in the urban center and 440,000 people in the larger metropolitan region. This presents challenges with relatively lower stroke volumes when compared to hospitals located in larger cities making resourcing of stroke neurologist even more challenging.

We believe that our study provides a frontier for future research aimed at improving the ASP process. Using our methodology as a foundation, stroke centers may identify their specific set of delay-causing sub-tasks at their respective facilities. Comprehensive stroke centers may utilize our approach to identify delay-causing sub-tasks in both IV thrombolysis and EVT treatment processes while primary stroke centers may focus on identifying optimization targets for IV thrombolysis at their centers. Based on their relevance and contextual conditions, potential strategies to optimize these delay-causing sub-tasks might include training sessions for efficient sub-task execution, parallel execution, or even considering the omission of certain non-value-adding sub-tasks.

Some limitations should be taken into consideration when interpreting the results of the current study. Firstly, it was not feasible to observe all patients arriving with an ASP activation, and the observations were limited to patients with an ETA exceeding 10 min. Additionally, the sample size was relatively small, which affected the observations for some sub-tasks that were recorded in low frequencies. Furthermore, the observations were limited to a specific time frame, from 7 am to 11 pm, and therefore, the sub-tasks identified in this study are limited to activities that occurred during the observation time frame. Finally, the study was conducted during the COVID-19 pandemic, which imposed health regulations and restrictions that may have slightly affected the AIS treatment process.

## 5. Conclusions

The aim of the current study was to identify the sub-tasks that are associated with AIS treatment and determine their impact on the overall ASP process. The study center recorded several sub-tasks related to ASP, highlighting the complexity of the ASP process. During phase 2, the study identified multiple individual or combined sub-tasks that caused delays in the treatment process, both when a non-stroke neurologist was involved or when the ASP was carried out after-hours. Moreover, the study identified several sub-tasks that were exclusively performed during specific treatment situations. These delay-causing sub-tasks resulted in longer CTNT, DNT, and CTGP time for both non-stroke neurologists and after-hours ASPs. Furthermore, when a non-stroke neurologist conducted the ASP, the DTGP time was longer.

The study provides valuable insights into the sub-tasks associated with AIS treatment and their impact on the overall ASP process. Although there are some limitations, our study offers a distinctive approach by identifying areas of concern and subsequently designing targeted quality improvement interventions that could cater to the unique challenges of individual stroke centers. Further research is required to validate the findings in different settings and establish a foundation for the generalizability of targeted interventions aimed at reducing treatment delays.

## Data availability statement

The original contributions presented in the study are included in the article/Supplementary material, further inquiries can be directed to the corresponding author.

## Ethics statement

The studies involving human participants were reviewed and approved by the Nova Scotia Health Authority Quality Improvement and Safety Council. Written informed consent from the patients OR patients legal guardian/next of kin was not required to participate in this study in accordance with the national legislation and the institutional requirements.

## Author contributions

GK: study design, data collection, data analysis, preparation of figures and tables, and preparation of drafts of manuscript. MK: data analysis, preparation of figures and tables, and preparation of drafts of manuscript. NK: study design, editing, formatting, funding the project, supervising the work, and revision of manuscript for intellectual content. GG: input to study design and revision of manuscript for intellectual content. All authors contributed to the article and approved the submitted version.
