# Supplementary material for: Optimizing acute stroke treatment process: insights from sub-tasks durations in a prospective observational time and motion study
Source: Front Neurol. 2023 Oct 27;14:1253065. doi: 10.3389/fneur.2023.1253065 (PMC10641836; doi:10.3389/fneur.2023.1253065)
Supplement: Supplementary file 1 [file Data_Sheet_1.docx]

Supplementary Material

Supplementary Table 1: Definitions of the sub-tasks observed during the study

| SUB-Tasks | Definition |
| --- | --- |
| 1. Arrival to Imaging (Prior to Imaging) | |
| - 1. *Triage and Registration* | Triage nurse and registration clerk completes the triage and registration |
| - - 1. *Triage* | Triage nurse completes triage of the patient with the information received from paramedics |
| - - 1. *Registration* | Registration clerk completes registration of the patient, it includes the time when the patient has to wait for the chart/bracelet |
| - 1. *Covid-19 Sample Collection* | ED nurse takes Covid-19 sample |
| - 1. *Laboratory Sample Collection* | ED nurse puts an IV-line for bloodwork and collects blood for laboratory |
| - 1. *Moving To ED Bed (for walk-ins and patients requiring stabilization)* | Moving the patient to ED bed or wheelchair; this can occur if the patient is transferred from another facility by Life Flight or if there is a need for a stabilization or if patient arrives by PV |
| - 1. *Stabilization* | Stabilizing patient before moving to Radiology department |
| - 1. *Share Patient’s Medical History* | The paramedics or emergency physician shares information about the event |
| - 1. *Neurological Evaluation* | Neurologist examines the patient and completes NIHSS |
| - 1. *Transport Imaging Department from Emergency Department* | The patient is transported to the radiology department |
| 1. Imaging Acquisition | |
| - 1. *Prepare Patient for Imaging* | CT technicians prepare the patient for the imaging session |
| - - 1. *Waiting for Scanner Availability* | After the arrival of radiology department, if the scanner is not available, patient waits for the scanner availability |
| - - 1. *Removing Jewelry and Hearings* | If the patient has any jewelry and/or hearings, removing them before starting imaging |
| - - 1. *Moving Patient to CT Table* | Moving the patient to CT table for imaging |
| - - 1. *Adjusting Scanning Machine* | Adjustment of scanning machine for the imaging |
| - 1. *CT Imaging* | Imaging session including interruptions during the imaging |
| - - 1. *Obtaining Brain Imaging* | Obtaining brain imaging (CT-CTA-CTP) |
| - - 1. *Interruptions During the Imaging* | Any interruption during the imaging for various reasons, such as dye problems |
| - 1. *Moving Patient from CT Table to Stretcher/ED Bed* | Moving patient back to stretcher or ED bed after imaging session completed |
| 1. Treatment Decision | |
| - 1. *Treatment Decision* | The neurologist and neurointerventionalist discuss the images, and the neurology physician decides about the treatment |
| - 1. *Revaluation* | If there is a need for repeating the neurological evaluation before the decision was made |
| - 1. *Consent Conversation* | The consent conversation, including contraindications list checking, takes a place before the decision of thrombolysis |
| - 1. *Additional Steps in Treatment Decision Making* | If there is a need for additional steps before treatment decision made. |
| - - 1. *Waiting for Laboratory Results* | If there is a need for checking for INR level before thrombolysis treatment, the neurologist waits for laboratory results |
| - - 1. *Using Point-of-Care INR Machine* | If there is a necessity to check INR level before admitting thrombolysis, the neurologist uses POC machine instead of waiting for lab results |
| 1. preparations for Thrombolysis | |
| - 1. *Return To ED and Move Patient To ED* | Patient needs to return to ED if there is a need |
| *4.1.1. Return To ED* | If there is a need to return to ED (patient-related reasons or during off-hours), the patient returns back to ED before/after the decision |
| *4.1.2. Moving Patient to ED Bed* | If the patient returns to ED, moving patient to ED bed |
| - 1. *ED Room Arrangement* | If the patient returns to ED and the room is not ready, patient waits for the room arrangement |
| - 1. *Prepare Patient and To Mix Thrombolysis* | Preparing patient before admitting thrombolysis |
| *4.3.1. Putting Second IV-Line* | The center requires two IV-Line before admitting thrombolysis; if the patient arrives without second IV-Line, putting another IV-Line is required |
| *4.3.2. Preparing Patient (Undressing)* | If the patient returns to ED, ED staff undresses the patient before anything else |
| *4.3.3. Treatment For High Blood Pressure/Glucose* | If the patient has either high blood pressure or high blood glucose, it needs to be reduced before admitting thrombolysis |
| *4.3.4. Mixing Thrombolysis* | Mixing thrombolysis before admitting it |
| 1. Preparations for EVT | |
| - 1. *Time To Prepare Angiosuite* | The angiosuite is prepared for the EVT procedure |
| - 1. *Time To Move Patient To Interventional Radiology Department* | The patient is transported to interventional radiology from the emergency department or radiology department waiting area for the EVT procedure |
| - 1. *Anesthesia Assessment Time* | The anesthesia physician assesses before the EVT |
| - 1. *Patient Preparation Time* | Preparing the patient for the EVT procedure |
| *5.4.1. Moving Patient to Angio Table* | The patient is put on the angiosuite table |
| *5.4.2. Preparing Patient* | The patient is prepared for the EVT procedure |
| *5.4.3. General Anesthesia Admission* | The general anesthesia was implemented if needed |

Supplement Table 2: Comprehensive overview of individual and combined sub-tasks observed during the study

| Sub-Tasks | Median Time  (IQR) | Routine working hours vs After-Hours | | | Stroke Neurologist vs Non-Stroke Neurologist | | |
| --- | --- | --- | --- | --- | --- | --- | --- |
|  |  | **Routine working hours** | **After-Hours** | **P value** | **Stroke Neurologist** | **Non-Stroke Neurologist** | **P value** |
| 1. **Arrival to Imaging (Prior to Imaging)** | | | | | | | |
| - 1. Time to Triage and Registration | 3 (2–7)  n=159 | 2 (1–7)  *n=71* | 4 (2–7)  *n=88* | 0.049 | 2 (2–6.75)  *n=44* | 3 (2–8)  *n=115* | 0.067 |
| - - 1. Time to Triage | 0 (0–0)  n=159 | 0 (0–0)  n=71 | 0 (0–0)  n=88 | 0.202 | 0 (0–0)  n=44 | 0 (0–0)  n=115 | 0.306 |
| - - 1. Time to Registration | 3 (2–6)  n=159 | 2 (1–7)  n=71 | 4 (2–6)  n=88 | 0.119 | 2 (2–6.75)  n=44 | 3 (2–6)  n=115 | 0.148 |
| - 1. Covid-19 Sample Collection Time | 1 (0–1)  n=84 | 1 (0–1)  *n=39* | 1 (0–1.5)  *n=45* | 0.215 | 0 (0–4)  *n=19* | 1 (0–1)  *n=65* | 0.481 |
| - 1. Laboratory Sample Collection Time | 3 (2.75–5)  n=102 | 3 (3–5)  *n=48* | 3.5 (2–5)  *n=54* | 0.833 | 4 (3–6)  *n=27* | 3 (2–5)  *n=75* | 0.173 |
| - 1. Time to Move to ED bed (for walk-ins and patients requiring stabilization) | 1 (1–1.5)  n=9 | 1 (1–1.5)  *n=5* | 1 (1–1.75)  *n=4* | 1 | 1 (1–2)  *n=3* | 1 (1–1.25)  *n=6* | 0.722 |
| - 1. Time for Stabilization | 33 (3–45)  n=3 | 24 (–)  *n=2* | 33 (–)  *n=1* | - | 45 (–)  *n=1* | 18 (–)  *n=2* | - |
| - 1. Time to Share Patient’s Medical History | 2 (1–2)  n=115 | 2 (1–2)  *n=48* | 2 (1–2)  *n=67* | 0.883 | 1.5 (1–2)  *n=28* | 2 (1–2)  *n=87* | 0.039 |
| - 1. Time for Neurological Evaluation | 3 (2–5)  n=156 | 3 (2–5)  *n=67* | 3 (2–5)  *n=89* | 0.453 | 3 (2–4)  *n=41* | 3 (2–5)  *n=115* | 0.523 |
| - 1. Time to Transport to Radiology Department from ED | 2 (2–2)  n=168 | 2 (2–2)  *n=72* | 2 (2–2)  *n=96* | 0.803 | 2 (2–2)  *n=44* | 2 (2–2)  *n=124* | 0.874 |
| 1. **Imaging Acquisition** | | | | | | | |
| - 1. Time to Prepare Patient for Imaging | 4 (4–5)  n=179 | 4 (3.5–5)  *n=77* | 4 (4–6)  *n=102* | 0.154 | 4 (4–5)  *n=45* | 4 (4–5)  *n=134* | 0.999 |
| - - 1. Time to Wait for Scanner Availability | 2 (1–4)  n=19 | 1 (1–3.5)  n=9 | 2.5 (1–5)  n=10 | 0.488 | 1 (1–2.5)  n=4 | 2 (1–5)  n=15 | 0.230 |
| - - 1. Time to Remove Jewelry and Hearings | 0.5 (0–1)  n=8 | 1 (–)  n=1 | 0 (0–1)  n=7 | - | 1 (–)  n=1 | 0 (0–1)  n=7 | - |
| - - 1. Time to Move Patient to CT Table | 2 (1–2)  n=179 | 2 (1–2)  n=77 | 2 (1 – 2)  n=102 | 0.577 | 2 (1–2)  n=45 | 2 (1–2)  n=134 | 0.938 |
| - - 1. Time to Adjust Scanning Machine | 3 (2–3)  n=179 | 3 (2–3)  n=77 | 3 (2–3)  n=102 | 0.429 | 3 (2–3)  n=45 | 3 (2–3)  n=134 | 0.906 |
| - 1. Time to CT Imaging | 6 (5–7)  n=182 | 6 (5–7)  *n=79* | 6 (5–8)  *n=106* | 0.232 | 6 (5–7)  *n=46* | 6 (5–8)  *n=139* | 0.326 |
| - - 1. Time to Obtain Brain Imaging | 6 (5–7)  n=182 | 6 (5–6)  n=79 | 6 (5–8)  n=103 | 0.447 | 5 (5–6)  n=46 | 6 (5–7)  n=136 | 0.338 |
| - - 1. Time for Interruptions during the Imaging | 3 (1.25–5)  n=20 | 3 (0.5–5.5)  n=5 | 3 (2–5)  n=15 | 0.727 | 3 (1–6)  n=3 | 3 (1.5–5)  n=17 | 1 |
| - 1. Time to Move Patient from CT Table to Stretcher/ED bed | 2 (2–3)  n=173 | 2 (2–3)  *n=73* | 2 (2–3)  *n=100* | 0.797 | 2 (1–2.75)  *n=44* | 2 (2–3)  *n=129* | 0.122 |
| 1. **Treatment Decision** | | | | | | | |
| - 1. Treatment Decision Time | 7 (4–11.25)  n=54 | 5 (2–10)  n=27 | 9 (6–12)  n=27 | 0.019 | 4.5 (0.5–9.75)  n=16 | 8 (5–12.25)  n=38 | 0.029 |
| - 1. Time to Revaluation | 3 (2.5–8)  n=9 | 5.5 (–)  n=2 | 3 (2–8)  n=7 | 0.661 | 1 (–)  n=1 | 3 (3–8)  n=8 | - |
| - 1. Time to Consent Conversation | 2 (2–3)  n=24 | 2 (2–2.75)  n=8 | 2 (1.25–3)  n=16 | 0.878 | 2 (2–2.5)  n=5 | 2 (1–3)  n=19 | 0.887 |
| - 1. Time for Additional Steps in Treatment Decision-Making | 12.5 (2.8 – 93.3)  n = 6 | 6 (2 – 94)  n=3 | 19 (3-93)  n=3 | 1 | (–)  *n=0* | 12.5 (2.8 – 93.3)  n=6 | - |
| - - 1. Time to Wait for Laboratory Results | 51.5 (9.3–90.8)  n=4 | 45 (–)  n=2 | 56 (–)  n=2 | 0.699 | (–)  *n=0* | 51.5 (9.3–90.8)  n=4 | - |
| - - 1. Time to Use Point-of-Care INR Machine | 3 (2–10)  n=3 | 6 (–)  *n=2* | 3 (–)  *n=1* | - | (–)  *n=0* | 3 (2–10)  *n=3* | - |
| 1. **Preparations for Thrombolysis** | | | | | | | |
| - 1. Time to Return ED and to Move Patient to ED Bed | 3 (3 – 4)  n=25 | 3 (2 – 4)  n=7 | 3 (3 – 4)  n=18 | 0.823 | 3.5 (2.25 – 7)  n=4 | 3 (3 – 4)  n=21 | 0.784 |
| - - 1. Time to Return ED | 2 (2–3)  n=25 | 3 (2–3)  n=7 | 2 (2–3)  n=18 | 0.183 | 2 (2–4.25)  n=4 | 2 (2–3)  n=21 | 1 |
| - - 1. Time to Move Patient to ED Bed | 1 (1–2)  n=20 | 1 (0–2.5)  n=5 | 1 (1–2)  n=15 | 0.600 | 2 (1–3)  n=3 | 1 (1–2)  n=17 | 0.290 |
| - 1. Time for ED Room Arrangement | 4 (1–6.5)  n=9 | 7 (–)  n=1 | 3 (1–5.5)  n=8 | - | (–)  *n=0* | 4 (1–6.5)  n=9 | - |
| - 1. Time to Prepare Patient and to Mix Thrombolysis | 7 (3–12)  n=37 | 4 (3 – 7)  n=19 | 11 (6.5 – 16)  n=18 | 0.004 | 3 (3 – 6)  n=10 | 8 (5 – 13)  n=27 | 0.011 |
| - - 1. Time to Put Second IV-Line | 2 (1–7)  n=11 | 2 (0–3)  n=3 | 2.5 (1.25–8.5)  n=8 | 0.414 | 1 (0–2)  n=3 | 3 (2–8.5)  n=8 | 0.066 |
| - - 1. Time to Prepare Patient (Undressing) | 5 (3–6)  n=17 | 5 (4.25–5.75)  n=4 | 5 (2–7)  n=13 | 0.734 | 4 (1–5)  n=3 | 5 (3.5–6.5)  n=14 | 0.257 |
| - - 1. Time for High Blood Pressure/High Blood Glucose Treatment | 9.5 (3.75–21.75)  n=6 | 4 (3–39)  n=3 | 12 (7–16)  n=3 | 0.663 | 3 (–)  n=1 | 12 (5.5–27.5)  n=5 | - |
| - - 1. Time to Mix Thrombolysis | 3 (3–4)  n=37 | 3 (3–3)  n=19 | 3 (2.75–4.25)  n=18 | 0.438 | 3 (2.75–3)  n=10 | 3 (3–5)  n=27 | 0.252 |
| 1. **Preparations for EVT** | | | | | | | |
| - 1. Time to Prepare Angiosuite | 34 (26–47.5)  n=25 | 27.5 (23.75–32.5)  *n=10* | 45 (33–50)  *n=15* | <0.001 | 27.5 (23.25–31.75)  *n=8* | 43 (31–50)  *n=17* | 0.012 |
| - 1. Time to Transport Patient to Interventional Radiology Department | 2 (1–3)  n=23 | 2 (1.25–2)  *n=12* | 2 (1–3)  *n=11* | 0.248 | 2 (1.25–2)  *n=8* | 2 (1–3)  *n=15* | 0.253 |
| - 1. Anesthesia Assessment Time | 2 (1–4)  n=15 | 2 (1–2.5)  *n=5* | 3 (1.75–5.25)  *n=10* | 0.150 | 2 (1–2)  *n=3* | 3 (1.25–4.75)  *n=12* | 0.248 |
| - 1. Patient Preparation Time | 12 (9.5–21.5)  n=25 | 14.5 (10.25–30)  *n=12* | 10 (9–19.5)  *n=13* | 0.201 | 14.5 (10.75–30)  *n=8* | 11 (9–19.5)  *n=17* | 0.220 |
| - - 1. Time to Move Patient to Angio Table | 2 (1–2)  n=24 | 2 (1.25–2.75)  n=12 | 1 (1–2)  n=12 | 0.157 | 2 (1.25–2)  n=8 | 1.5 (1–2.75)  n=16 | 0.624 |
| - - 1. Time to Prepare Patient | 10 (8–18.5)  n=25 | 11.5 (8.25–27.25)  n=12 | 9 (8–16.5)  n=13 | 0.314 | 13 (8.75–27.25)  n=8 | 9 (8–16.5)  n=17 | 0.200 |
| - - 1. Time for General Anesthesia Admission | 9 (6–9)  n=3 | 9 (–)  n=2 | 6 (–)  n=1 | - | 9 (–)  n=1 | 7.5 (–)  n=2 | - |
